# Supplementary material for: Formulation of silages from spent mushroom substrates of Pleurotus ostreatus and Lentinula edodes: Organoleptic properties, phenolic content, in vitro digestibility, gas production and ruminal kinetics
Source: PLoS One. 2025 Sep 5;20(9):e0331467. doi: 10.1371/journal.pone.0331467 (PMC12412943; doi:10.1371/journal.pone.0331467)
Supplement: S1 Raw image — Picture was taken using a cellphone camera (iPhone model 14) with a handheld Mineralight® Multi-D Lamp, UVSL-25, corresponding to Fig 2. (PDF) [file pone.0331467.s001.pdf]

Mycotoxin determination by thin layer chromatography (TLC)

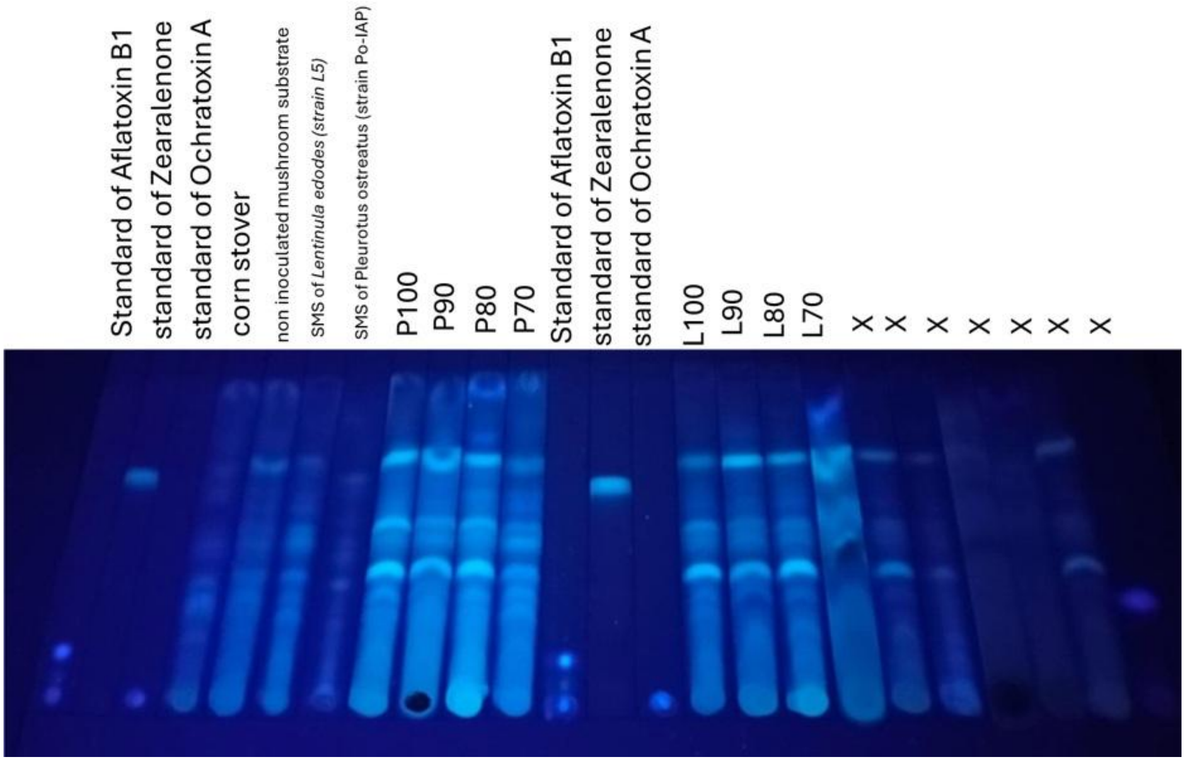

Picture was taken using a cellphone camera (iPhone model 14) using a handheld Mineralight® Multi-D Lamp, UVSL-25.
