# Supplementary material for: Formulation of silages from spent mushroom substrates of Pleurotus ostreatus and Lentinula edodes: Organoleptic properties, phenolic content, in vitro digestibility, gas production and ruminal kinetics
Source: PLoS One. 2025 Sep 5;20(9):e0331467. doi: 10.1371/journal.pone.0331467 (PMC12412943; doi:10.1371/journal.pone.0331467)
Supplement: S2 Table — (DOCX) [file pone.0331467.s002.docx]

**S2_Table. *In vitro digestibility of dry matter* of micro-silages pH value and ammoniacal nitrogen (NH_3_-N) from SMS of *Lentinula edodes* L5 and *Pleurotus ostreatus IAP***

| **Inclusion x SMS** | **IVDMD (%)** | | **pH** | | **NH3-N (mg/dL)** | |
| --- | --- | --- | --- | --- | --- | --- |
|  | **Mean** | **standard deviation** | **Mean** | **standard deviation** | **Mean** | **standard deviation** |
| **L100** | 58.6 cd | 14.198 | 7.08 | 0.097 | 32.9 | 3.1 |
| **L90** | 61.5 bc | 3.317 | 6.97 | 0.085 | 31.2 | 2.44 |
| **L80** | 67.6 ab | 13.193 | 6.88 | 0.095 | 31.2 | 5.14 |
| **L70** | 73.5 a | 12.777 | 6.81 | 0.115 | 30.8 | 2.88 |
| **P100** | 53.4 d | 15.854 | 6.99 | 0.111 | 31.8 | 3.7 |
| **P90** | 67.4 ab | 19.19 | 6.88 | 0.142 | 31.5 | 2.27 |
| **P80** | 69.9 a | 13.962 | 6.79 | 0.16 | 31.2 | 4.82 |
| **P70** | 74. 0 a | 12.523 | 6.72 | 0.186 | 30.5 | 3.01 |
| **SMS** |  | | | | | |
| **L** | 65.30 | 13.00 | 6.93a | 0.14 | 31.53 | 3.62 |
| **P** | 66.17 | 17.33 | 6.84b | 0.18 | 31.29 | 3.59 |
| **Inclusion** |  | | | | | |
| **100** | 55.96c | 15.21 | 7.03a | 0.11 | 32.38a | 3.44 |
| **90** | 64.47b | 14.03 | 6.92b | 0.12 | 31.39ab | 2.35 |
| **80** | 68.77b | 13.58 | 6.8c | 0.14 | 31.19ab | 4.96 |
| **70** | 73.73a | 12.60 | 6.7d | 0.16 | 30.68b | 2.94 |
| **P value Inclusion x SMS** | 0.0096 | | 0.8954 | | 0.2924 | |
| **P value inclusion** | <0.0001 | | <0.0001 | | 0.0001 | |
| **P value SMS** | 0.4642 | | <0.0001 | | 0.3785 | |
