# Supplementary material for: Formulation of silages from spent mushroom substrates of Pleurotus ostreatus and Lentinula edodes: Organoleptic properties, phenolic content, in vitro digestibility, gas production and ruminal kinetics
Source: PLoS One. 2025 Sep 5;20(9):e0331467. doi: 10.1371/journal.pone.0331467 (PMC12412943; doi:10.1371/journal.pone.0331467)
Supplement: S3 Table — (DOCX) [file pone.0331467.s003.docx]

**S3_Table. maximum volume of gas produced (Vmax), gas production rate (S), lag phase (L) after *in vitro* fermentation of micro-silages from SMS of *Lentinula edodes* L5 and *Pleurotus ostreatus IAP*.**

| **Inclusion x SMS** | **Vmax (mL/ g DM)** | | **S (h-1)** | | **L (h)** | |
| --- | --- | --- | --- | --- | --- | --- |
|  | **Mean** | **standard deviation** | **Mean** | **standard deviation** | **Mean** | **standard deviation** |
| **L100** | 140.8 g | 18.3 | 0.023 | 0.0025 | 6.00 b | 2.26 |
| **L90** | 208.8 e | 20.61 | 0.033 | 0.0023 | 1.33 g | 0.9 |
| **L80** | 259.7 c | 29.79 | 0.043 | 0.0058 | 2.48 ef | 1.238 |
| **L70** | 306.5 a | 21.12 | 0.053 | 0.0627 | 2.83 de | 1.567 |
| **P100** | 113.3 h | 19.13 | 0.022 | 0.003 | 9.20 a | 4.202 |
| **P90** | 155.2 f | 12.57 | 0.03 | 0.0066 | 1.45 fg | 1.19 |
| **P80** | 227.7 d | 18.84 | 0.039 | 0.0053 | 3.63 cd | 1.453 |
| **P70** | 274.4 b | 20.11 | 0.04 | 0.0046 | 4.17 c | 1.602 |
| **SMS** |  | | | | | |
| **L** | 228.93a | 65.71 | 0.038a | 0.03 | 3.16b | 2.34 |
| **P** | 192.66b | 65.09 | 0.033b | 0.01 | 4.61a | 3.73 |
| **Inclusion** |  | | | | | |
| **100** | 127.04d | 23.18 | 0.023c | 0.003 | 7.60a | 3.72 |
| **90** | 181.97c | 31.83 | 0.032b | 0.01 | 1.39c | 1.05 |
| **80** | 243.71b | 29.55 | 0.041a | 0.01 | 3.06b | 1.46 |
| **70** | 290.47a | 26.09 | 0.046a | 0.04 | 3.50b | 1.71 |
| **P value Inclusion x SMS** | <0.0001 | | 0.1726 | | <0.0001 | |
| **P value inclusion** | <0.0001 | | <0.0001 | | <0.0001 | |
| **P value SMS** | <0.0001 | | 0.0116 | | <0.0001 | |
