# Supplementary material for: Formulation of silages from spent mushroom substrates of Pleurotus ostreatus and Lentinula edodes: Organoleptic properties, phenolic content, in vitro digestibility, gas production and ruminal kinetics
Source: PLoS One. 2025 Sep 5;20(9):e0331467. doi: 10.1371/journal.pone.0331467 (PMC12412943; doi:10.1371/journal.pone.0331467)
Supplement: S4 Table — (DOCX) [file pone.0331467.s004.docx]

**S4_Table. Total volatile fatty acid (VFA) concentration, acetic acid (%), propionic acid (%) and butyric acid (%) after *in vitro* fermentation of micro-silages from SMS of *Lentinula edodes* L5 and *Pleurotus ostreatus IAP*.**

| **Inclusion x SMS** | **Total volatile fatty acid (VFA) (mM)** | | **Acetic acid (%)** | | **Propionic acid (%)** | | **Butyric acid (%)** | |
| --- | --- | --- | --- | --- | --- | --- | --- | --- |
|  | **Mean** | **standard deviation** | **Mean** | **standard deviation** | **Mean** | **standard deviation** | **Media** | **Desv. est.** |
| **L100** | 34.35 | 8.36 | 65.21 | 1.03 | 24.23 ab | 1.03 | 10.56 | 0.7 |
| **L90** | 46.02 | 12.51 | 62.66 | 0.84 | 25.01 a | 0.61 | 12.32 | 0.51 |
| **L80** | 51.2 | 11.13 | 61.7 | 1.56 | 24.95 a | 1.01 | 13.35 | 0.71 |
| **L70** | 54.8 | 15.29 | 61.91 | 3.62 | 23.70 ab | 4.4 | 14.39 | 1.41 |
| **P100** | 33 | 8.16 | 66.35 | 1.74 | 23.14 b | 1.48 | 10.51 | 73 |
| **P90** | 41.78 | 12.81 | 63.48 | 1.4 | 24.66 ab | 0.94 | 11.85 | 0.78 |
| **P80** | 50.36 | 14.06 | 61.66 | 1.63 | 24.87 a | 1.16 | 13.47 | 0.69 |
| **P70** | 53.22 | 15.56 | 61.31 | 1.95 | 24.82 ab | 1.07 | 13.87 | 1.03 |
| **SMS** |  | | | | | | | |
| **L** | 46.59 | 14.17 | 62.87 | 2.48 | 24.47 | 2.36 | 12.66 | 1.67 |
| **P** | 44.59 | 14.98 | 63.20 | 2.61 | 24.37 | 1.36 | 12.43 | 1.57 |
| **Inclusion** |  | | | | | | | |
| **100** | 33.67c | 8.18 | 65.78a | 1.52 | 23.68b | 1.38 | 10.54d | 0.70 |
| **90** | 43.90b | 12.68 | 63.07b | 1.21 | 24.84a | 0.80 | 12.09c | 0.69 |
| **80** | 50.78ab | 12.52 | 61.68c | 1.58 | 24.91a | 1.07 | 13.41b | 0.69 |
| **70** | 54.01a | 15.24 | 61.61c | 2.89 | 24.26ab | 3.21 | 14.13a | 1.25 |
| **P value Inclusion x SMS** | 0.2645 | | 0.0655 | | 0.0414 | | 0.1849 | |
| **P value inclusion** | <0.0001 | | <0.0001 | | 0.0057 | | <0.0001 | |
| **P value SMS** | 0.3126 | | 0.1194 | | 0.719 | | 0.0638 | |
