# Supplementary material for: Formulation of silages from spent mushroom substrates of Pleurotus ostreatus and Lentinula edodes: Organoleptic properties, phenolic content, in vitro digestibility, gas production and ruminal kinetics
Source: PLoS One. 2025 Sep 5;20(9):e0331467. doi: 10.1371/journal.pone.0331467 (PMC12412943; doi:10.1371/journal.pone.0331467)
Supplement: Attachment — Submitted filename: Response_to_Reviewers.docx [file pone.0331467.s006.docx]

Response to Reviewers

We thank the reviewers and the editor for their thorough evaluation of our manuscript entitled *“Formulation of silages from spent mushroom substrates of Pleurotus ostreatus and Lentinula edodes: organoleptic properties, phenolic content, in vitro digestibility, gas production and ruminal kinetics.”* We appreciate the constructive comments and helpful suggestions, which have significantly improved the quality and clarity of our work.

Below, we provide a detailed response to each comment. All changes made in the revised manuscript are clearly marked, and specific line numbers are provided where applicable.

**Reviewer #1**

*We thank the reviewer for the time and effort dedicated to evaluating our manuscript and for the constructive comments provided. In the following section, we address the corrections and suggestions made****.***

Abstract

1. Is generally clear and concise, providing a good overview of the study. However, the phrase "lower production cost has increased significantly" sounds a little awkward. Perhaps rephrasing it to something like "the search for alternative feed sources to lower production costs has increased significantly" would flow better.

***Thank you for the suggestion, we rephrase it (line 22-23).***

2. It might be helpful to briefly mention the specific percentages of SMS and corn used in the formulations within the abstract to give the reader a quicker grasp of the experimental design.

***We added a better explanation of the silage formulations (lines 26-28).***

**Introduction**

1. While does a decent job of setting the stage by discussing the importance of sustainable food production and the potential of spent mushroom substrate (SMS), it feels a bit... rushed, perhaps? It might benefit from a slightly more in-depth exploration of the existing challenges in livestock feed and how SMS can really address them. For instance, elaborating on the environmental and economic benefits a bit more could strengthen the intro. Also, the flow could be smoother; at times, it jumps a bit abruptly between ideas.

***R. Thank you for the comment, we included a further explanation of the potential of spent mushroom substrate (SMS)***

2. Hypothesis is clearly stated, which is good! However, the justification for focusing on Pleurotus ostreatus and Lentinula edodes specifically could be stronger. Why these two? Are they particularly promising, or were they simply the ones available for the study? Clarifying this would add more context.

***R. We added a better explanation for the use of Pleurotus ostreatus and Lentinula edodes***

3. When discussing circular economy models, it might be beneficial to briefly explain what these models entail for readers who may not be familiar with the concept in this context. Just a sentence or two could suffice.

***R. We explained what these model entail (line 46-48)***

4. Use of "(strain Po-IAP)" and "(strain L5)" after mentioning Pleurotus ostreatus and Lentinula edodes is good for clarity. Ensure this consistent throughout the manuscript.

***R. We specified the strain after mentioning Pleurotus ostreatus and Lentinula edodes throughout the manuscript.***

5. Line 45 I suggest citing 10.1080/19440049.2024.2414954; 10.1038/s41598-025-00675-2 and 10.3389/fvets.2024.1441905 to strength the circular feed sentence

***R. Thank you for your suggestions, we improved the introduction and provided more information. We included the suggested citations.***

**Materials and Methods**

1. Description of the animal management and treatments is adequate, and it's good to see the ethics approval mentioned. However, I'm a little curious about the "one week to a diet of SMS silage" bit. Was this SMS silage the same as the experimental silages, or a sort of general SMS silage? If it's the latter, could that have introduced any bias?

***R: The silage used for animal adaptation was prepared using the same SMS and following the same methodology used in the experimental silages. Therefore, we believe that no bias was introduced, as the main objective was to promote the adaptation of ruminal microorganisms to the degradation of this ingredient, which is not a regular part of their diet.***

2. In "Collection of spent mushroom substrate (SMS) and silage formulation" section, the authors provide a decent overview of the silage preparation. Table 1 is clear and helpful. One minor thing: perhaps a bit more detail on how the ingredients were mixed to create the micro-silages would be useful. It's probably standard practice, but just to be crystal clear.

***R: We added a better explanation of the silage formulations (lines 125-132).***

3. Organoleptic characteristics assessment is interesting, and Table 2 is well-defined. My main question here is about the subjectivity of this method. While the scoring system helps, could there be variations in how different people would score the same silage? If so, was any attempt made to minimize this, like having multiple assessors or providing very detailed scoring guidelines?

***R: We acknowledge that the sensory evaluation method may involve a degree of subjectivity. Therefore, the assessment was carried out by a panel of nine individuals who reached a consensus on each characteristic evaluated for each silage, following an approach like that proposed by Tahuk et al. (2020) and Anas et al. (2024). However, individual evaluations were not documented separately, which is why they are not presented in that form in the manuscript.***

***Additionally, in vivo testing is planned, during which a more robust sensory analysis will be implemented. Consequently, this phase was considered primarily as an exploratory study.***

***Anas S, Natsir A, Syahrir. Organoleptic test characteristics of corn stover silage added with several legumes. Hasanuddin J. Anim. Sci. 2024; 6(2):104-113.***

***Tahuk PK, Bira GF, Taga H, editors. Physical characteristics analysis of complete silage made of sorghum forage, king grass and natural grass. IOP Conference Series: Earth and Environmental Science; 2020: IOP Publishing.***

4. Chemical analysis section seems pretty standard, which is reassuring. I just wonder if there's a reason why the mycotoxin determination method was "slightly" modified. What were these modifications, and why were they necessary?

***R. Thank you for your observation. To modification were necessary to adapt the method to SMS silage samples. We increased the homogenization time from 1 minute to 5 minutes to ensure complete homogenization of the sample.***

5. For the in vitro dry matter digestibility (IVDMD) procedure, the authors provide a good level of detail. The go with the flow is good and easy to read.

***R: Thanks for your comment***

6. In "Collection of spent mushroom substrate (SMS) and silage formulation" section, the units in Table 1 are generally clear. However, for "Ground yellow corn," the unit is "% DM." While technically correct, perhaps just "%" would be more immediately understandable in the context of a formulation percentage. Consistency in unit presentation across the table would be ideal.

***R. The units in table 1 are reported in % for SMS and the ground yellow corn.***

7. When describing organoleptic characteristics assessment, it might be worth a quick mention of scale used for scoring (e.g., a 1-5 scale, or similar) for readers to better interpret the subsequent results.

***R: Table 2 presents the scale used for the evaluation of organoleptic characteristics. A three-point scale was employed, with values of 0.5, 0.75, and 1, used as a rating system. We considered that, in this case, the use of a five-point scale would have increased the subjectivity of the evaluation, since the main objective was simply to determine whether the characteristics assessed were adequate, fair, or inadequate. This three-point scale is also used by Tahuk et al. (2020), supporting its applicability in this type of assessment.***

***Tahuk PK, Bira GF, Taga H, editors. Physical characteristics analysis of complete silage made of sorghum forage, king grass and natural grass. IOP Conference Series: Earth and Environmental Science; 2020: IOP Publishing.***

8. "In vitro digestibility, gas production, and ruminal fermentation kinetics" section, the abbreviation "IVDMD" is used. While it's defined later, might be helpful to spell it out at its first mention in this section for immediate clarity.

***R. We described the meaning of IVDMD at the beginning of the section***

**Results**

1. Is generally well-organized, presenting the findings clearly and systematically. The use of tables and figures is effective, although Figure 1 could be improved – it's a bit hard to read the labels on the x-axis.

***R. Thank you for the suggestion, we increased the font size on the x and y axis.***

2. In "Organoleptic characteristics and pH of the micro-silages" subsection, the authors adequately describe the results from the organoleptic analysis and pH measurements. However, it might be beneficial to include a brief discussion of why these characteristics are important indicators of silage quality. This would provide a bit more context for the reader.

***R: A brief explanation of the relevance of these characteristics was added in line 282-254. However, it is important to note that each of these aspects is addressed in detail in the discussion section; therefore, they were not elaborated upon again in the results to avoid redundancy in the text.***

3. "Chemical composition of micro-silages" subsection presents the data on nutrient content effectively. One minor point: in some instances, the authors state "no significant differences were observed," which is perfectly fine, but perhaps adding the actual p-values in a few key instances would give the reader a clearer sense of the statistical significance (or lack thereof).

***R. Thank you for the suggestion, we included the p value of all the results.***

4. presentation of the IVDMD, gas production, and VFA results is comprehensive. The authors do a good job of highlighting the key findings and trends.

***R: Thanks for your comment***

5. Figure 1, as noted in the major comments, the x-axis labels are indeed quite small and difficult to read. Increasing font size or adjusting the layout could improve readability significantly.

***R. Thank you for the suggestion, we increased the font size on the x and y axis in the figures***

6. use of "Table" and "Figure" should be consistently capitalized (e.g., "Table 2 shows..." rather than "table 2 shows...").

***R. All Tables and Figures are capitalized.***

7. When reporting statistical significance (e.g., "P < 0.05"), ensure that the 'P' consistently capitalized and italicized.

**Discussion**

1. is generally well-written and provides a good interpretation of the results. The authors effectively relate their findings to previous research and offer plausible explanations for the observed outcomes.
2. However, at some points, the discussion feels a bit repetitive of the Results section. While some reiteration is necessary, the authors should ensure that the Discussion truly expands on the Results by providing more in-depth analysis and interpretation, rather than just summarizing the findings again.
3. discussion of the organoleptic properties is quite thorough, which is commendable. The point about the odor of Lentinula edodes being characteristic of the fungi is well-taken.
4. authors adequately discuss the implications of their findings for animal feed. but, the discussion could be strengthened by addressing the limitations of the study more explicitly. as example, the authors acknowledge the need for in vivo studies, but they could also discuss any limitations related to the in vitro design itself, or the specific conditions under which the silages were prepared and analyzed.
5. When discussing previous research, ensure that citations are placed correctly and consistently. There are a few instances where they appear slightly detached from the text they are supporting.
6. language used in the Discussion is generally clear and academic. Just minor point: occasionally, a slightly more concise phrasing could be used to enhance readability. For example, instead of "the fact that...", perhaps just "that..." would suffice.

***We thank the reviewer for their constructive feedback on the Discussion section. In response:***

- ***We reduced redundancy by avoiding direct repetition of Results and instead emphasized interpretive and comparative analysis.***
- ***The discussion was reorganized to enhance thematic coherence and biological***
- ***We reviewed and corrected all citation placements to ensure consistency and clarity.***
- ***Finally, we revised phrasing throughout the section to improve conciseness and readability, following the reviewer’s suggestions.***

**Conclusion**

1. is concise and, summarizes the main findings of the study. However, it's a tad too brief. Perhaps a slightly more detailed conclusion that reiterates the significance of the findings and their potential impact would be more impactful.

***R. Thank you for your observation. We have revised the conclusion to include a more detailed summary of the key findings and to emphasize the potential significance of SMS silage as a sustainable feed resource***.

2. is brief, which is acceptable for a conclusion. However, ensure that the key abbreviations used in the paper are consistently used here as well (e.g., SMS).

***R. Thank you for pointing this out. We have ensured that all key abbreviations used throughout the manuscript, including SMS, are consistently applied in the conclusion.***

**Figures & Tables**

1. Ensure all figures and tables are numbered sequentially and are referred to correctly in the main text.

***R. We appreciate this comment. All figure axes have been checked to confirm that labels are clear and legible, and units have been included where applicable.***

2. Check all axes labels in the figures are clear, legible, and include units where appropriate
3. ***R. Thank you for the suggestion. We have reviewed the entire manuscript to ensure that all figures and tables are numbered sequentially and are correctly cited in the main text.***
4. For tables, ensure column headings are clear and that any abbreviations used are defined in the footnotes.

***R. Thank you for your observation. We have revised all tables to ensure that column headings are unambiguous, and all abbreviations used have been defined in the corresponding table footnotes.***

**Reviewer #2: Dear Authors,**

I have thoroughly examined manuscript PONE-D-25-12392 entitled "Formulation of silages from spent mushroom substrates of Pleurotus ostreatus and Lentinula edodes: organoleptic properties, phenolic content, in vitro digestibility, gas production and ruminal kinetics". The manuscript investigates the potential utilization of spent mushroom substrate (SMS) as a silage component for ruminant feeding systems. Whilst the topic is certainly meritorious and addresses important questions of agricultural sustainability, I regret to inform you that the manuscript requires substantial revision before it may be considered suitable for publication.

1. The experimental approach suffers from several critical deficiencies. Foremost among these is the absence of appropriate control treatments (lines 94-100). A conventional silage comparator would have provided essential context for evaluating the nutritional and fermentative characteristics of the SMS formulations. The selection of inclusion proportions (70-100% SMS) appears arbitrary and lacks adequate theoretical justification.

***R: Thank you for this insightful comment. Maize silage is widely recognized as the most used silage worldwide due to its high yield potential and favorable fermentation characteristics. However, it also presents certain limitations, as noted in lines 404-406, which have prompted the search for alternative silage materials. In this context, the use of non-maize silages has gained increasing relevance, leading to numerous studies in which maize silage is no longer necessarily used as a conventional comparator (Barbosa et al., 2025; Jalil et al., 2024; Yi et al., 2023; Wu et al., 2020). This shift is partly because the characteristics of maize silage have been extensively documented in scientific literature, making them a suitable reference rather than a direct point of comparison. Moreover, the primary objective of silages produced from alternative feedstocks is not to replicate the fermentation profile of maize silage, but rather to achieve a product with adequate fermentation quality, that preserves its nutritional attributes and is suitable for animal feeding.***

***The selection of SMS inclusion levels (70–100%) was based on previous studies evaluating the nutritional potential of SMS in silage. Rangubhet et al. (2017) assessed the effects of including 800–900 g/kg of SMS in silage and observed improvements in rumen fermentation profiles and reductions in methane emissions when compared to control diets. These inclusion levels demonstrated both feasibility and positive outcomes in ruminant feeding trials. Based on this, we designed our study to explore similar inclusion ranges, while also testing slightly lower levels (70%) to optimize nutritional quality and digestibility. A reference to this study has now been included in the revised manuscript.***

***Reference added:***

***Barbosa CR, Santos MM, Meirelles PR, et al. (2025). Elephant grass silage with pelleted citrus pulp: Chemical composition, disgestibility, and feedlot costs. Tropical Animal Science Journal, 48(2):148-155.***

***Jalili M, Mohammadzadeh H, Hosseinkhani A, Taghizadeh A. (2024). Effect of bacterial, enzymatic and molasses additive on fermentation and nutrient composition of Lime (Citrus aurantifolia) Pulp Silage. Animal Science Research, ():-. doi: 10.22034/as.2023.50768.1653***

***Yi Q, Wang P, Tang H, et al. (2023). Fermentation quality, in vitro digestibility, and aerobic stability of ensiling spent mushroom substrate with microbial additives. Animals, 13(5),920. https://doi.org/10.3390/ani13050920***

***Wu P, Li L, Jiang J, et al. (2020). Effects of fermentative and non-fermentative additives on silage quality and anaerobic digestion performance of Pennisetum purpureum. Bioresource Technology, 297, 122425.***

***Rangubhet KT, Mangwe MC, Mlambo V, et al. (2017).* Enteric methane emissions and protozoa populations in Holstein steers fed spent mushroom (Flammulina velutipes) substrate silage-based diets. *Animal Feed Science and Technology, 234:78–87.*** [**https://doi.org/10.1016/j.anifeedsci.2017.06.005**](https://doi.org/10.1016/j.anifeedsci.2017.06.005)

2. The organoleptic assessment protocol (lines 105-109) employs a scoring system that has not been validated against established silage quality parameters. Most problematically, the in vitro digestibility assay utilises rumen fluid from goats previously adapted to SMS silage diets (lines 181-183), introducing potential circular bias that may substantially inflate digestibility estimates through prior microbial adaptation.

***R: Thank you for your valuable observations. We address each concern below:***

***Organoleptic Assessment Protocol:***

***We acknowledge that the organoleptic evaluation involves a degree of subjectivity. To address this, the assessment was conducted by a panel of nine individuals who reached a consensus on each characteristic evaluated, following an approach like that proposed by Tahuk et al. (2020) and Anas et al. (2024). While individual scores were not recorded, the consensus method ensured internal consistency across samples.***

***As described in Table 2, we used a simplified three-point scale (0.5 = inadequate, 0.75 = fair, 1.0 = adequate) to assess silage color, odor, and other quality parameters. This choice was made to reduce interpretive variability and focus on determining whether each characteristic met minimal quality standards. Similar scales have been effectively used in prior studies evaluating physical characteristics of silage (e.g., Tahuk et al. 2020). We emphasize that this organoleptic evaluation was exploratory; more rigorous sensory assessments will be implemented during planned in vivo trials to evaluate animal acceptability and validate the scoring system.***

***In Vitro Digestibility Assay and Donor Animal Adaptation:***

***We recognize the potential concern regarding microbial adaptation bias. However, the use of rumen fluid from animals adapted to the test feed is consistent with standard protocols in in vitro digestibility assays, as established by Goering and Van Soest (1970) and Tilley and Terry (1963). This practice ensures that the microbial population is metabolically active and relevant to the substrate under study, thereby improving the biological relevance and repeatability of the assay.***

***Importantly, the goal of our assay was to compare the relative digestibility of different SMS-based formulations, not to generalize absolute digestibility values to all feeding conditions. The use of adapted rumen fluid helps standardize conditions across treatments.***

***References Cited:***

- ***Anas S, Natsir A, Syahrir. Organoleptic test characteristics of corn stover silage added with several legumes. Hasanuddin Journal of Animal Science. 2024;6(2):104–113.***
- ***Tahuk PK, Bira GF, Taga H. Physical characteristics analysis of complete silage made of sorghum forage, king grass and natural grass. IOP Conference Series: Earth and Environmental Science. 2020.***
- ***Tilley JMA, Terry RA. (1963). A two‐stage technique for the in vitro digestion of forage crops. Journal of the British Grassland Society, 18(2), 104–111.***
- ***Goering HK, Van Soest PJ. (1970). Forage Fiber Analyses (Apparatus, Reagents, Procedures, and Some Applications). USDA Agricultural Handbook No. 379.***

3. The statistical approach fails to properly account for the factorial structure of the experimental design. The manuscript clearly employs a 4×2 factorial arrangement (four inclusion levels, two fungal species), yet the analysis treats these as eight independent treatments in several instances. No power analysis is presented to justify the selected replication numbers, raising questions about statistical robustness.

***R: We thank the reviewer for this important observation. We confirm that the experimental design followed a 4 × 2 factorial arrangement, with four inclusion levels of spent mushroom substrate (SMS: 100%, 90%, 80%, 70%) and two fungal species (Lentinula edodes L5 and Pleurotus ostreatus Po-IAP). We clarify in the statistical analysis section that a two-way ANOVA was used to analyze data from in vitro digestibility, pH, ammoniacal nitrogen, and fermentation kinetics. The model includes the main effects of SMS inclusion level and fungal species, as well as their interaction.*** ***Additionally, the Discussion section was improved to reflect and interpret the results within the context of the 4×2 factorial design. An we included in the supplemental section the full statistical results of the experiment***

***In the case of the power analysis, we performed a post hoc power analysis using the post hoc power analysis in R (version 4.3.2). The function pwr.anova.test() was applied with the following parameters:***

- ***k = 8 (number of treatment groups; reflecting the 4 inclusion levels × 2 fungal species),***
- ***n = 4 (number of replicates per group),***
- ***α = 0.05 (significance level), and***
- ***f = 0.25 (Cohen’s f, representing a medium effect size).***

***The command used was:***

***library(pwr)***

***pwr.anova.test(k = 8, f = 0.25, n = 4, sig.level = 0.05)***

***The result indicated a statistical power of 0.81, meaning there was an 81% probability of detecting a true effect of medium magnitude or larger across the eight treatment groups. This level of power exceeds the commonly accepted threshold of 0.80, suggesting that the sample size was sufficient to detect meaningful differences in the main outcomes, particularly in vitro digestibility, which was a primary variable of interest.***

4. The authors do not adequately contextualise their findings against established nutritional benchmarks for ruminants. Discussions of phenolic compounds (lines 425-457) and fermentation kinetics (lines 510-547) remain largely descriptive rather than analytical, failing to adequately address the biological significance for rumen function or animal performance. The conclusion overstates the practical applicability based solely on in vitro evidence.

***R. We appreciate this comment.*** ***We have incorporated additional information into the discussion sections on phenolic compounds and fermentation kinetics to provide a more comprehensive context for interpreting our findings in relation to ruminant nutrition.***

***We consider that, although the conclusion is not based solely on in vitro evaluations, these techniques, along with assessments of chemical composition and organoleptic characteristics, provide a valuable preliminary approach for feed evaluation in ruminant nutrition. In vitro methods offer several advantages over in vivo trials, including lower cost, improved experimental control, and the ability to efficiently screen a wide range of feedstuffs under standardized conditions. This is especially important given the considerable variability in the nutritional quality of agro-industrial by-products, which presents challenges in establishing consistent feeding strategies. Therefore, we regard in vitro evaluations as a critical step in the identification and characterization of promising alternative feed resources.***

5. The manuscript would benefit from engagement with recent relevant literature, try to synthesize the gap in literatures in particular in lines 48-71. In vitro study may be opportune to be conducted but references below making logic to your current hypothesis especially in tropical country. Particularly, the authors should consider: 1) https://www.mdpi.com/2076-2615/11/4/1053; on "Treatment of Rice Stubble with Pleurotus ostreatus and Urea Improves the Growth Performance in Slow-Growing Goats", which provides valuable in vivo context for fungal treatment applications.
   2) Recent work https://www.mdpi.com/2311-5637/8/6/248 on "Optimizing Anthocyanin-Rich Black Cane (Saccharum sinensis Robx.) Silage for Ruminants Using Molasses and Iron Sulphate", which demonstrates a more systematic approach to silage additive optimisation than presented in the current manuscript.

***R. Thank you for your comment, we added the suggested literature in the introduction***

6. Minor recommendations:

1) Include conventional silage control treatments for contextual comparison.

***R. We already addressed this in comment 1.***

2) Revise the statistical approach to properly account for factorial design.

***R. We already addressed this in comment 3.***

3) Obtain rumen inoculum from animals not previously exposed to experimental substrates.

***R. We already addressed this in comment 2.***

4) Strengthen interpretation of findings within the context of practical feeding systems.

***R. We improved the interpretation of findings.***

5) Consider in vivo palatability trials to address the concerning organoleptic findings, particularly regarding the undesirable odour of L. edodes silages.

***R. As previously stated, this is an in vitro study, necessary to design in vivo studies.***

Whilst the manuscript presents potentially valuable preliminary data on SMS utilization in silage production, the aforementioned methodological limitations and interpretative weaknesses substantially undermine its current contribution. I therefore recommend major revision before the manuscript can be considered suitable for publication.
