# Supplementary material for: Formulation of silages from spent mushroom substrates of Pleurotus ostreatus and Lentinula edodes: Organoleptic properties, phenolic content, in vitro digestibility, gas production and ruminal kinetics
Source: PLoS One. 2025 Sep 5;20(9):e0331467. doi: 10.1371/journal.pone.0331467 (PMC12412943; doi:10.1371/journal.pone.0331467)
Supplement: Attachment — Submitted filename: Response_to_Reviewers_auresp_2.docx [file pone.0331467.s007.docx]

Response to Reviewers

We thank the reviewer and the editor for their thorough evaluation of our manuscript entitled *“Formulation of silages from spent mushroom substrates of Pleurotus ostreatus and Lentinula edodes: organoleptic properties, phenolic content, in vitro digestibility, gas production and ruminal kinetics.”* We appreciate the constructive comments and helpful suggestions, which have significantly improved the quality and clarity of our work.

Below, we provide a detailed response to each comment. All changes made in the revised manuscript are clearly marked, and specific line numbers are provided where applicable.

**Reviewer #1**

We thank the reviewer for the time and effort dedicated to evaluating our manuscript and for the constructive comments provided. In the following section, we address the corrections and suggestions made**.**

Materials and Methods

1. Include conventional silage control treatments for contextual comparison. This could help provide a better baseline for how your SMS formulations perform.

***R: Thank you for your suggestion. Maize silage is widely recognized as the most used silage worldwide due to its high yield potential and favorable fermentation characteristics. However, it also presents certain limitations, as noted in lines 404-406, which have prompted the search for alternative silage materials. In this context, the use of non-maize silages has gained increasing relevance, leading to numerous studies in which maize silage is no longer necessarily used as a conventional comparator (Barbosa et al., 2025; Jalil et al., 2024; Yi et al., 2023; Wu et al., 2020). This shift is partly because the characteristics of maize silage have been extensively documented in scientific literature, making them a suitable reference rather than a direct point of comparison. Moreover, the primary objective of silage produced from alternative feedstocks is not to replicate the fermentation profile of maize silage, but rather to achieve a product with adequate fermentation quality, that preserves its nutritional attributes and is suitable for animal feeding.***

***Reference added:***

***Barbosa CR, Santos MM, Meirelles PR, et al. (2025). Elephant grass silage with pelleted citrus pulp: Chemical composition, disgestibility, and feedlot costs. Tropical Animal Science Journal, 48(2):148-155.***

***Jalili M, Mohammadzadeh H, Hosseinkhani A, Taghizadeh A. (2024). Effect of bacterial, enzymatic and molasses additive on fermentation and nutrient composition of Lime (Citrus aurantifolia) Pulp Silage. Animal Science Research, ():-. doi: 10.22034/as.2023.50768.1653***

***Yi Q, Wang P, Tang H, et al. (2023). Fermentation quality, in vitro digestibility, and aerobic stability of ensiling spent mushroom substrate with microbial additives. Animals, 13(5),920. https://doi.org/10.3390/ani13050920***

***Wu P, Li L, Jiang J, et al. (2020). Effects of fermentative and non-fermentative additives on silage quality and anaerobic digestion performance of Pennisetum purpureum. Bioresource Technology, 297, 122425.***

2. You should revise the statistical approach to properly account for the factorial design. It seems like in some parts, the analysis might be treating things as separate when they are part of a larger experimental setup.

***R. As you indicated, the data on in vitro gas production and chemical composition come from a single experiment. However, because of their disparate data structures and research goals, the data could not be examined using the same statistical model:***

- ***Since each analysis sought to characterize the nutritional profile of each silage treatment separately and independently, one-way ANOVA was used to analyze the chemical composition and phenolic content. Since treatment was the primary factor and we were interested in the properties of the formulated silages, we did not consider the impact of the interaction between the levels of inclusion or the type of SMS.***
- ***On the other hand, data on ruminal kinetics, digestibility, and in vitro gas production were gathered over time and necessitated a factorial approach (4 levels of SMS inclusion × 2 mushroom species). To take into consideration the main effects as well as the interaction effects of species and inclusion levels on dynamic fermentative outcomes, these variables were examined using a 2-way ANOVA under a 4 × 2 factorial design.***

3. When getting rumen inoculum, it would be good to obtain it from animals that haven't been exposed to the experimental substrates before. This could help avoid any potential biases in the results.

***R. Thank you for the comment. We recognize the potential concern regarding microbial adaptation bias. However, the use of rumen fluid from animals adapted to the test feed is consistent with standard protocols in in vitro digestibility assays, as established by Goering and Van Soest (1970) and Tilley and Terry (1963). This practice ensures that the microbial population is metabolically active and relevant to the substrate under study, thereby improving the biological relevance and repeatability of the assay.***

***Importantly, the goal of our assay was to compare the relative digestibility of different SMS-based formulations, not to generalize absolute digestibility values to all feeding conditions. The use of adapted rumen fluid helps standardize conditions across treatments.***

***References Cited:***

- ***Tilley JMA, Terry RA. (1963). A two‐stage technique for the in vitro digestion of forage crops. Journal of the British Grassland Society, 18(2), 104–111.***
- ***Goering HK, Van Soest PJ. (1970). Forage Fiber Analyses (Apparatus, Reagents, Procedures, and Some Applications). USDA Agricultural Handbook No. 379.***

Discussion

4. Try to strengthen the interpretation of your findings within the context of practical feeding systems. Making it clearer how this research applies to real-world scenarios would be beneficial.

***R. Thank you for your suggestion to strengthen the interpretation of our findings within practical feeding systems. We included a paragraph in the discussion section to ensure the applicability of our research to real-world animal production contexts (Line 681-688).***

5. You might want to consider in vivo palatability trials to address the concerning organoleptic findings, especially regarding the undesirable odor of *L. edodes silages. This could provide valuable insights into how animals actually perceive the feed.*

***R. We appreciate this comment. The present study is an in vitro study the provided a first approach of the possible use of silage formulated with SMS from L. edodes and P. ostreatus. The organoleptic evaluation indicated an undesirable and potentially unpalatable odor in the L. edodes silages, which may negatively influence animal acceptance***

***We addressed this limitation and included in the discussion section the necessity of in vivo palatability trials to evaluate the animal response and feed intake behavior (Line 453-455)***.
